# The role of digital twins in P4 medicine: A paradigm for modern healthcare

**DOI:** 10.1038/s41746-025-02115-x

**Published:** 2025-12-01

**Authors:** Frank Emmert-Streib, Seppo Parkkila, Reinhard Laubenbacher, Arto Mannermaa, Leroy Hood, Olli Yli-Harja

**Affiliations:** 1 Faculty of Information Technology and Communication Sciences, Tampere University, Tampere, Finland; 2 Faculty of Medicine and Health Technology, Tampere University, Tampere, Finland; 3 Department of Clinical Chemistry, Fimlab Laboratories Ltd, Tampere University Hospital, Tampere, Finland; 4 Department of Medicine, University of Florida, Gainesville, FL USA; 5 Biobank of Eastern Finland, Kuopio University Hospital, Kuopio, Finland; 6 Institute of Clinical Medicine, Pathology and Forensic Medicine, and Multidisciplinary Cancer Research Community, University of Eastern Finland, Kuopio, Finland; 7 Institute for Systems Biology, Seattle, WA USA

**Keywords:** Health care, Medical research, Engineering

## Abstract

P4 medicine (Predictive, Preventive, Personalized, and Participatory) offers a comprehensive approach to personalized healthcare, emphasizing both the transition from disease to wellness and the importance of preventive care. In this perspective, we propose a novel extension to P4 medicine. Specifically, we argue that combining P4 medicine with digital twins (DTs) introduces capabilities that elevate it far beyond its current scope. While P4 medicine provides a conceptual framework grounded in medical principles, digital twins offer a complementary framework for methodological realization. Together, these concepts form a synergistic pairing, interfacing through individual patients and their corresponding data. Furthermore, we emphasize that digital twins represent a new paradigm—not merely a method—characterized by four critical features: explainability, intervenability, learnability, and diversability. We believe that this integration unlocks capabilities that go beyond the reach of traditional bioinformatics and systems biology approaches.

## Introduction

Systematic approaches to medicine and healthcare evolve over time in response to new knowledge, data, and technological and methodological advances. For instance, the success of Evidence-Based Medicine (EBM) is closely tied to randomized controlled trials (RCTs), which enable the investigation of causal effects of interventions such as treatments and medications. This, in turn, helps identify the most effective options for patient care^1,2^. An extension of population-based EBM is P4 medicine (predictive, preventive, personalized, and participatory)^3^. Building on advancements from the Human Genome Project (HGP), which led to novel measurement technologies such as next-generation sequencing, P4 medicine leverages high-throughput data through bioinformatics and especially systems biology across various modalities, including genomics, transcriptomics, metabolomics, and proteomics, to develop patient-specific models. It also transforms the role of patients from passive recipients to active participants in their healthcare^3,4^.

In this perspective, we propose a novel extension to P4 medicine. Specifically, we argue that the practical implementation of P4 medicine cannot rely solely on data-driven methods, even when leveraging diverse data sources. Instead, it must be complemented by a dynamic, model-driven approach, as enabled by digital twins. While P4 medicine offers a conceptual framework rooted in medical principles, digital twins provide a methodological framework for practical realization. This synergy offers a concrete path toward realizing the full potential of P4 medicine in clinical practice.

To illustrate this, in the following, we start by introducing the concept of digital twins followed by a discussion of four key features supporting P4 medicine. Briefly, these features relate to: explainability, intervenability, learnability and diversability. To highlight the transformative impact of these key features on P4 medicine, we provide an illustrative example demonstrating their interplay in the context of brain health and neurodegeneration (BHN).

## Digital twin as part of an ecosystem

The idea of a digital twin (DT) originated in manufacturing^5^, and most applications to date are found in that field and in engineering domains^6^. In recent years, however, the concept has gained significant interest in other scientific areas, including climate research, urban development, and the health sciences^7–9^. In simple terms, a digital twin is an idealized concept: a digital representation of a real-world object that closely mirrors its physical counterpart, or physical twin, continuously updated over time^10–12^. While this digital representation utilizes computer-based simulations it is more than an ordinary simulation model^13^.

Importantly, there are two key points that often cause confusion and hinder a proper understanding of digital twins. First, a digital twin is not a specific method, but rather a paradigm—a conceptual framework that guides how data, models, and simulations are integrated to dynamically represent a real-world system. Second, a DT is not functional in isolation; it must operate within a broader infrastructure, referred to as a digital twin system (DTS), which provides the necessary data pipelines, user interfaces, and decision-making tools.

As a paradigm, the DT is defined by a set of core features, such as data integration, personalization, and virtual intervention capabilities, but its practical realization varies across domains. This flexibility means that there is no single implementation of a DT; instead, diverse methodological approaches may all qualify, depending on the context. For example, in robotics, DTs are typically implemented using control-theoretic models and sensor-driven feedback loops, which differ significantly from the models used in biomedical applications. Consequently, methods are not directly transferable between domains. Rather, it is the underlying conceptual framework of digital twins that is adapted to fit the specific needs and constraints of each field.

To accomplish such tasks, a digital twin cannot simply be a standalone simulation model. Instead, it operates within an ecosystem, i.e., a broader interconnected system comprising various components and technologies. This system includes the digital representation of the physical entity, as well as the integration of data sources, analytics tools, and user interfaces, all working together to deliver comprehensive insights and functionalities^10,12^. Referred to as a digital twin system (DTS)^14^, the DTS also provides clarity on the role of AI in connection with digital twins. This will be further discussed in later sections.

Practical applications of digital twins in medicine are still at an early stage; however, their benefits have been realized and widely discussed (see Table 1). The contributions shown in the table are forward-looking, offering perspectives, comments, and outlooks on possible applications of digital twins. They range from addressing individual diseases like cancer and multiple sclerosis to broader implications for personalized medicine and drug development.

**Table 1:** Examples for digital twins in medicine and healthcare

Subject	Reference	Article type	Content
Cardiology	^36^	C	Concepts in a digital twin model of the heart (2021).
Clinical oncology	^37^	R	Integrating medical imaging with mechanism-based, tissue-scale mathematical modeling (2022).
T1 diabetes	^16^	A	Closed-loop system of glucose metabolism functioning as an artificial pancreas (2019).
T2 diabetes	^38^	A	DT for chronic kidney disease (CKD) identification utilizing generalized metabolic fluxes (GMF) (2024).
Multiple sclerosis	^39^	R	Improve diagnosis, monitoring, therapy, and enabling prevention of disease progression (2021).
Viral infection	^40^	P	Personalized computer simulations of infection could allow more effective treatments (2021).
Immune system	^41^	P	Roadmap for building a prototype of an immune digital twin (2022).
Oncology	^42^	A	DT integrating mutation, miRNA expression, imaging, and clinical data for kidney and colon cancer (2024).
Healthcare	^43^	R	DT multi-agent simulation, monitoring the patient’s personal health indicators and their development (2025).
Mental health	^44^	A	DT for the detection of mental health conditions through dialog-based natural language processing systems (2024).
Critical Care	^45^	A	Optimize critical care workflows to monitor physical entities in the Critical Care Unit (2025).
Drug development	^46^	P	Drug Development Digital Twin (DDDT) for drug discovery, testing and repurposing (2022).

A: Article, P: perspective, C: comment, R: review.

One of the earliest clinically tested applications of digital twins in the medical domain is the *artificial pancreas* model for treating patients with type I diabetes^15^. The artificial pancreas is a mathematical model that simulates the glucose metabolism of a target patient. The model operates in a closed-loop system, receiving real-time blood glucose levels from a sensor worn by the patient to predict the necessary insulin dose. If a deviation is detected, a pump attached to the patient administers the appropriate amount of insulin^16^.

Despite this success story, a clear connection between P4 medicine or precision medicine and digital twins is currently lacking. To address this gap, we begin by discussing several key features of digital twins that advance P4 medicine.

## Transformative features of digital twins for P4 medicine

For P4 medicine, digital twins (DTs) offer several distinct features that go beyond traditional analytical approaches. As we will be discuss below, these enhanced functionalities provide the following capabilities:

1. Explainability
2. Intervenability
3. Learnability
4. Diversability

In simple terms, these capabilities can be summarized as follows: (1) Explainability: DTs are built on mechanistic models with structures interpretable in biological and medical terms, facilitating meaningful discussions among clinicians and approaches to mechanistic causality. (2) Intervenability: DTs can serve not only as predictive models but also as tools for virtual interventions, such as assessing the effects of different medications and lifestyle interventions. (3) Learnability: DTs evolve into personalized patient models by integrating new data over time. (4) Diversability: By leveraging uncertainties, DTs can create ensembles, enabling preventive and participatory measures, such as early diagnosis. We use the terms Explainability, Intervenability, Learnability, and Diversability for these capabilities to underscore their extent and significance. Each term encapsulates a distinct and crucial dimension of how digital twins function and contribute to advancing P4 medicine.

Each of these features will be discussed in detail in the following sections to provide a deeper understanding of their transformative potential for P4 medicine.

### Explainable models

**Fig. 1:**
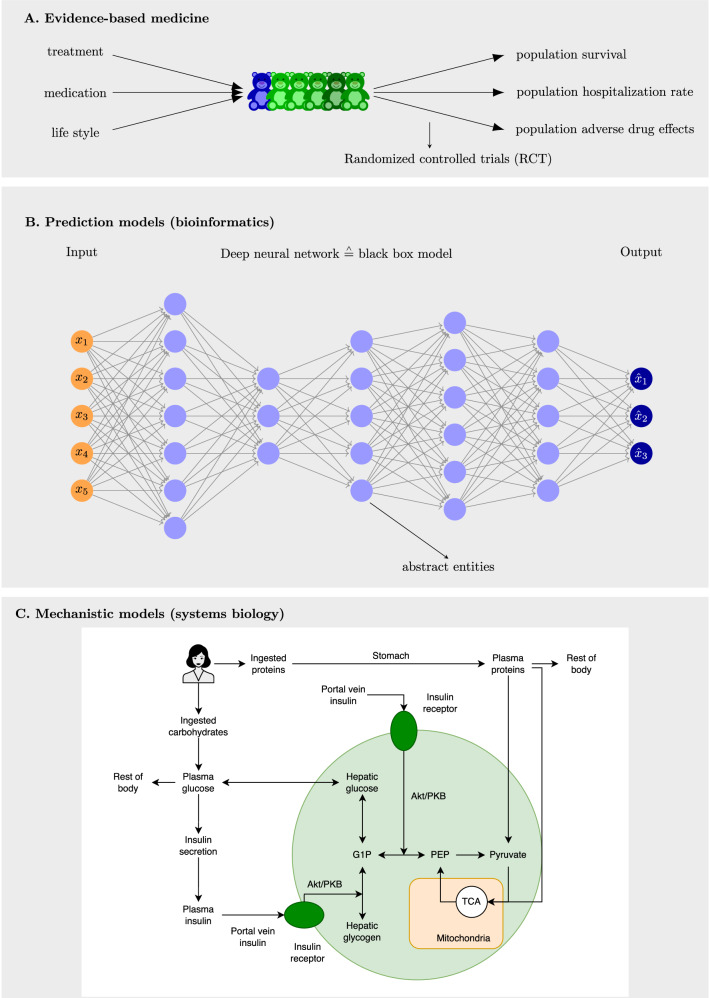
Explainability. **A** Evidence-based medicine derives population-level insights through randomized controlled trials (RCTs). **B** Prediction models in bioinformatics frequently utilize black-box models, e.g., those based on deep learning. **C** Models in systems biology offer mechanistic descriptions of molecular processes, resulting in explainable models. The example shows a model of human metabolism of diet response^18^.

A key emphasis of P4 medicine is on predictions. General prediction models have been developed in computational and systems biology, resulting in a large arsenal of methods capable of handling various data types and characteristics. Recently, deep neural networks (DNNs) have been added to this list due to their improved predictive abilities^17^. In Fig. 1B, we show an example of such a DNN in the form of a feedforward neural network with five hidden layers (in blue), distinguishing it from classical artificial neural networks having just one hidden layer. A characteristic of general predictive models, including deep neural networks (DNNs), is that their internal components—such as the hidden neurons (highlighted in blue in Fig. 1B)—typically lack meaningful biological interpretation. This opaqueness is why such models are commonly referred to as black-box models. When applied in bioinformatics, this lack of interpretability means that the resulting models do not offer biological insight and are therefore considered non-explainable.

In contrast to general predictive models, digital twins are built on mechanistic models that explicitly represent underlying biological and physiological processes. This distinction is illustrated in Fig. 1C, which shows a model of human metabolism and dietary response^18^, compared to a deep neural network (DNN)-based prediction model in Fig. 1B. A key advantage of mechanistic models is that each component has a direct biological interpretation, making the model’s structure and behavior interpretable within the biological domain. In contrast, the internal features of a DNN lack this transparency, making them difficult—or even impossible—to interpret in biological terms. This challenge has given rise to the field of explainable AI (XAI)^19^, which seeks to improve the interpretability of AI-driven predictions, particularly in high-stakes areas such as medicine and healthcare where trust and understanding are essential^20^.

The mechanistic nature of a digital twin (DT) establishes its explainability, providing understandable and interpretable insights. This form of explainability can be regarded as a passive attribute—emerging naturally from the model’s structure and its alignment with biological or physical processes. In contrast, the next key feature of a DT lies in its active capabilities: the ability to be manipulated or adjusted to simulate specific conditions, interventions, or scenarios.

### Virtual interventions: Testing What-If scenarios

The second key feature of a digital twin is its ability to simulate virtual interventions. In a medical context, an intervention may involve administering a drug that alters protein binding behavior through conformational changes, thereby affecting the structure of regulatory networks^21^. Traditionally, such effects can only be observed by administering the drug to a patient. In contrast, a DT allows its underlying mechanistic model to be modified, enabling the simulation of interventions and the generation of new quantitative predictions. This makes it possible to evaluate the impact of various therapeutic options virtually. As a result, multiple drugs can be systematically tested in silico, yielding estimates of their efficacy and associated uncertainties—before any real-world administration.

**Fig. 2:**
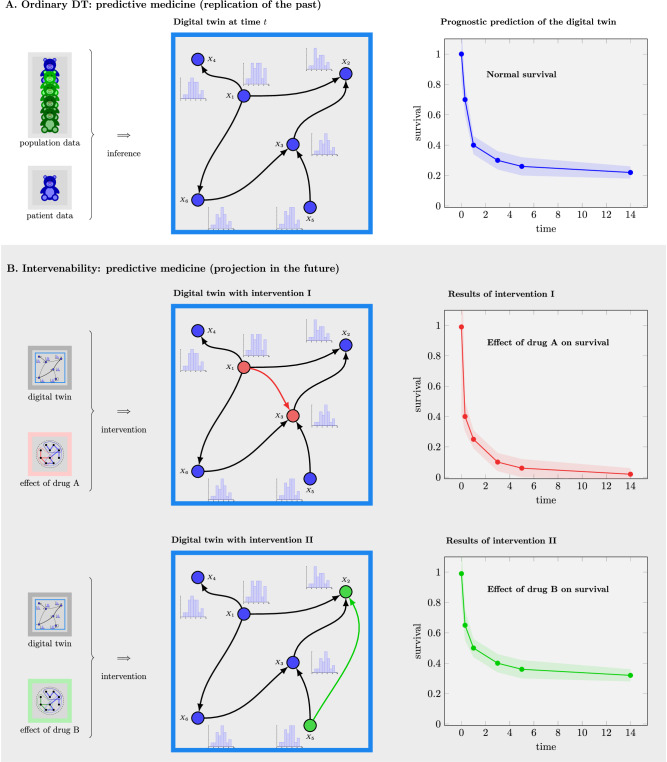
Intervenability of digital twins. **A** digital twin can not only be used to generalize predictions from training data to unseen samples (see A) but for testing virtual interventions (VIs) (see B). The example in (**B**) illustrates how two drugs affect protein binding, resulting in a modified model structure. This structural change alters the dynamic behavior of the digital twin, leading to different predictions, such as the drug’s efficacy, which ultimately impacts patient survival.

An example of virtual drug testing is shown in Fig. 2. The top section of the figure illustrates a digital twin and its resulting prognostic predictions about a patient. In Fig. 2B, two drugs are tested, leading to structural changes in the regulatory network (highlighted in color). These changes occur either through direct binding to proteins or by binding to enzymes, thereby affecting enzymatic functions. Such interventions result in altered dynamic behavior within the mechanistic models and manifest in changed predictions. These predictions can then be compared and utilized by clinicians to support informed decision-making thereby advancing predictive medicine.

We would like to emphasize that introducing interventions, such as changes to the network structure of a digital twin, enables the exploration of “What-If" scenarios and their effects on predictions. This capability is made possible by the mechanistic model underlying the digital twin. In contrast, black-box prediction models, due to their abstract and opaque nature (see Fig. 1B), do not support meaningful structural modifications. As a result, the ability to test virtual interventions is a distinctive feature of mechanistic models, which is not present in black-box models.

Another important aspect of virtual interventions is their potential to promote participatory medicine. This is due to the fact that the results of such analyses can be discussed collaboratively between the clinician and the patient, potentially using interactive visualizations on a dashboard to support joint decision-making about treatment options. This approach can help translate medical jargon into practical terms understandable by a layperson.

On a research-related note, virtual interventions offer additional benefits. For instance, they can help narrow down the search space of potential drugs by identifying a smaller subset that demonstrates favorable prognostic effects on patient survival. This, in turn, supports efficient experimental design, reduces experimental costs, and lessens the need for labor-intensive laboratory work. It also offers the possibility that two or more drugs may be studied simultaneous for their effects on relevant disease-perturbed networks.

### Learnability of digital twins

**Fig. 3:**
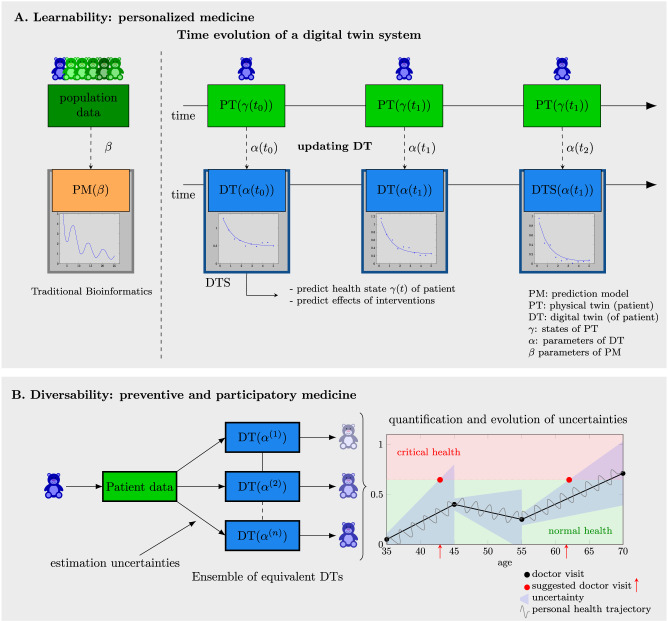
Learnability and diversability of digital twins. **A** The learnability of a digital twin allows for predictive capabilities that surpass those of traditional bioinformatics approaches. Left: Traditional (static) bioinformatics approach typically based on population data. Right: Time evoluation of a digital twin system periodically updated with new patient data. **B** The diversability of a digital twin can lead to an ensemble of equivalent DTs. This can be utilized for estimating doctor visits, e.g., when confidence intervals cross a critical threshold (see red dashed line). This approach supports preventive and participatory medicine by facilitating early diagnosis and timely treatment of diseases.

The third feature of a digital twin is learnability. This is depicted in Fig. 3A. In contrast to traditional bioinformatics or systems biology approaches, a DT undergoes a series of updates over time allowing to adjust its parameters by utilizing new data, e.g., from the patient, providing up to date information about the health status. Ideally, this could involve a continuous flow of data, but in practice, it may be confined to discrete time points as permitted by health standards.

Limiting factors of the update frequency are physiological and regulatory. Specifically, the number of invasive procedures, such as biopsies, is severely limited because each such procedure carries risks and causes distress to the patient. On the other hand, non-invasive procedures, such as those based on imaging technologies, alleviate this concern to some extent but currently cannot be performed continuously. Furthermore, ethical regulations ensure that all treatment and care options conform to current legislation.

We would like to highlight that updating a digital twin makes the model not only more accurate but also more personalized, either to a particular patient or a disease state, over time. Hence, the learnability of a DT leads to a personalized digital twin that has the potential to avoid population-based medicine issue that treat every patient from a particular disease-group in the same way.

For the methodological realization of personalized digital twins, we would like to highlight physics-informed neural networks (PINNs) as an example of applying artificial intelligence (AI)^22^. Specifically, PINNs can be used to estimate the parameters of dynamical systems that serve as mechanistic models, leveraging deep learning—a cornerstone of current AI approaches. This framework allows for the continuous updating of model parameters over time, potentially incorporating a Bayesian approach through Bayesian PINNs (B-PINNs)^22^.

One immediate benefit of digital twins in personalized medicine is its ability to support continued learning over an extended period. Studies in machine learning have demonstrated that this approach enhances model learnability due to an increased sample size. Furthermore, collecting data prior to disease onset enables the creation of a reference digital twin representing a patient’s healthy state. This facilitates patient-specific comparisons following the onset of a disease, providing more accurate insights into changes in biological processes while accounting for individual variability in biological activity.

From a practical point of view, to fully realize the potential of digital twins, it is essential to ensure that established models remain accessible beyond the duration of specific funding periods. Currently, standard methodological approaches do not always guarantee this continuity, despite widespread recognition of the importance of data availability for reproducible research^23^. In the context of digital twins, this would mean enabling them to learn and evolve over decades rather than years, incorporating data from multiple projects instead of being limited to a single one. This approach could establish robust population-level digital twins for specific disease subtypes, which could then serve as a starting point for personalized digital twins by adjusting the parameters to reflect patient-specific data. Ultimately, this approach enables the development of disease- and patient-specific digital twins, moving away from a singular, “one-size-fits-all" model.

### Diversability

The fourth feature of a digital twin is related to the fact that estimates of a digital twin from data are always accompanied by uncertainties, e.g., due to noise or measurement errors. Practically, this means the estimated parameters (*α*) of a digital twin cannot be uniquely identified but multiple consistent estimates are possible. Conceptually, this leads to an ensemble of equivalent digital twins for a patient, each having different but consistent parameters with the data available. This ensemble or population of DTs can be utilized for the quantification of uncertainties of resulting predictions, e.g., in form of confidence intervals. Technically, such estimates can be achieved via Monte Carlo (MC) dropout for prediction models resulting in point predictors^24^. Figure 3B illustrates this by showing one of multiple potential health trajectories (in black) for a patient, with uncertainties increasing over time (in blue) until new data are obtained, for example, during a doctor’s visit.

An example of utilizing the uncertainties in digital twin models is predicting when a patient should schedule a new doctor visit. Such visits are recommended when the health trajectory, along with its associated uncertainties, crosses a critical threshold (represented by the red dashed line in Fig. 3B). If utilized this way, this approach contributes to preventive and participatory medicine by enabling early diagnosis and timely treatment of diseases.

We call this feature “diversability" because it highlights that a patient’s health or disease state is not sharply defined but exists as a spectrum of similar yet distinct configurations. This implies that a patient’s long-term fate cannot be uniquely determined, even with extensive data. Therefore, ongoing health evaluations and regular check-ups are essential for accurate assessment and optimal patient-specific decision-making regarding treatment options.

## Example: Brain health and mental disorders

For studying mental health, we briefly exemplify a digital twin approach. By leveraging diverse datasets, such as proteomic, lipidomic, metabolomic, phenomic, and electronic health records, digital twins can model brain homeostasis, track cognitive decline, and identify critical biomarkers^25^. The framework begins with a mathematical model of brain function, integrating key biological subsystems relevant to mental health disorders. A digital population of hypotheses is then generated to predict diverse patient trajectories. Crucially, real-world patient data is used to instantiate and continuously refine personalized digital twins, improving their predictive accuracy over time. Furthermore, advanced analytical methods, including AI and machine learning, help uncover functional links between molecular processes and clinical outcomes. This approach enables more precise diagnostics, early risk assessment, and tailored interventions, ultimately enhancing personalized mental healthcare and deepening our understanding of psychiatric and neurodegenerative disorders, such as Alzheimer’s disease, Attention-deficit/hyperactivity disorder (ADHD), and depression.

Recently, a digital twin for brain homeostasis has provided remarkable insights into a comprehensive model of Alzheimer’s disease^25^. This model integrates top-down physiological results and differential equations of brain behavior and equilibria together with bottom-up clinical and omic data. Preliminary integrated omic analyses with this digital twin suggest that Alzheimer’s disease is a problem of brain metabolism and cholesterol clearance abnormalities and not just amyloid.

A second example of brain abnormalities focuses on the assessment of 25 different cognitive features (e.g. reaction speed, depth of field, memory, etc) with a digital health assessment called brain HQ^25^. This approach demonstrated that for the average adult cognition rises to a maximum in the late 20s and inevitably declines there after for most individuals. The brain HQ also has the ability to restore lost cognitive functions by a series of different brain exercises. Michael Merzenich, et al. were to demonstrate in individuals in their 80s that lost cognitive functions could substantially restored them with appropriate exercises—indicating the plasticity of brain cognition. They showed that practicing brain HQ throughout life exercises one’s brain effectively, just as we do with our hearts, and these exercises can insure maintaining cognitive effectiveness and delay the onset of depression or even neurodegenerative diseases^26,27^. The placement of these data in an effective brain digital twin model will add powerfully to capacity to make effective virtual digital twin predictions for individuals.

## Potential misconceptions

Digital twins (DTs) in engineering often rely on assumptions that would be unrealistic, or even unethical, when applied in a medical context. Crucially, such assumptions are not required for a medical system to qualify as a digital twin. In the following, we take a devil’s advocate perspective by addressing common misconceptions about digital twins and clarifying what is (and is not) essential for their proper functioning and application.

### Digital twins require real-time data

A critical requirement for digital twins is the availability of time-series or longitudinal data. In engineering domains, such data are often readily obtained through continuous sensor monitoring. In contrast, medical applications typically lack real-time access to most data modalities, with the exception of relatively simple variables such as heart rate. Consider, for instance, gene expression profiling of a tumor. Obtaining such data requires a biopsy, which is an invasive procedure involving physical risks. Therefore, it is neither practical nor ethically permissible to conduct biopsies daily or even hourly. This limitation highlights the challenges of implementing digital twins in clinical contexts.

Notably, the time scale of relevant changes in health or disease states is of critical importance. Since such changes in medical problems typically occur gradually over prolonged periods, often months or years, rather than instantaneously, gathering new data at similar intervals should be sufficient and efficient for model updates.

One powerful approach to obtaining near real-time data for the whole body is the realization that “blood is a window into global health and disease”^28^. One powerful example of this vision is the large-scale use of organ-specific blood proteins^29,30^. These are transcripts present in one organ at about the 90% level whose proteins are expressed in blood. We analyzed in this context the transcriptomes of 25 different organs. For example, the brain has more that 600 brain-specific transcripts that for the most part are highly localized in the brain. In a healthy brain, only a small fraction of these brain-specific blood proteins can be detected in the blood. This number increases enormously with serious brain injury or disease—even permitting one to localize site(s) at which the blood brain barrier has been lost. Thus the organ-specific proteins for 25 organs permit one to follow changes of state in many different organs simultaneously across the body.

### Digital twins should represent the entire body

While David Gelernter, credited with envisioning digital twins in his forward-looking book Mirror Worlds^31^, describes a digital twin on a societal level, such models are not inherently restricted by scale or scope. For example, the artificial pancreas model discussed earlier^16^, despite its limited scope, clearly demonstrates the value of digital twins with a focus far smaller than the entire body.

In principle, digital twins could one day model entire societies, encompassing individuals along with their social and economic interactions. However, in the near term, it is more realistic to view digital twins as focused models with a targeted scope. This more modest perspective does not diminish their potential impact in the context of P4 medicine; rather, it enables the development of practical and actionable tools within a foreseeable timeframe.

### Digital twins do not rely on AI

Since digital twins constitute a paradigm rather than a specific method, their relationship to artificial intelligence (AI) may not be immediately apparent. However, as outlined in^32^, the integration of AI within digital twin systems can be understood across at least six distinct stages of involvement.

1. DT model creation
2. DT model updating
3. Generative modeling
4. Data analytics
5. Predictive analytics
6. Decision making

The utility of artificial intelligence begins with the construction of the digital twin (DT) model itself. One promising direction is symbolic regression, which aims to infer the governing equations of dynamical systems directly from data. Recent developments that integrate deep learning have substantially improved the effectiveness of symbolic regression, offering both high predictive accuracy and interpretability in modeling complex system behavior^33,34^. Building on this foundation, AI techniques such as physics-informed neural networks (PINNs) support the ongoing calibration and refinement of DTs by estimating parameters in the underlying models. This dynamic updating capability enhances the digital twin’s fidelity and responsiveness to real-world conditions.

Another example of AI integration is generative modeling, particularly through the combination of large language models (LLMs) with knowledge graphs (KGs). This synergy offers a powerful framework for integrating heterogeneous data sources, extracting actionable insights, and supporting personalized healthcare. LLMs can process and extract information from unstructured text, such as clinical notes, research articles, and medical records, to populate and continuously update KGs. In turn, KGs provide an ontology by a structured and interpretable representation of medical knowledge by connecting entities such as diseases, drugs, symptoms, and treatments, enabling more informed and context-aware reasoning.

Beyond generative modeling, AI plays a central role in data analytics and predictive analytics, which represent the core analytical functions of a digital twin. Data analytics involves the integration and interpretation of heterogeneous sources, including external observations (e.g., patient data from the physical twin) and internal simulations generated through AI-driven generative models. This combination enables continuous monitoring, anomaly detection, and system state estimation. Predictive analytics builds upon these insights to forecast future system behavior, evaluate potential scenarios, and assess the outcomes of hypothetical interventions. These predictive capabilities, in turn, form the basis for decision making, where the DT supports informed recommendations^35^. In this way, AI empowers digital twins to function not merely as passive representations but as dynamic, adaptive systems capable of supporting complex decision-making.

### The Scientific Validity of Digital Twins

The final misconception we address concerns the scientific validity of digital twins. One argument raised against digital twins, particularly in the context of virtual interventions, is that they are merely simulations. This implies that the results of such virtual interventions may not accurately reflect the behavior of the real physical or biological twin. It is important to emphasize that predictions made by any model, whether digital twins or others, should not be confused with experimental results. Instead, predictions from these models must be tested to determine if they align with experimental data. Only if models cannot be falsified through such tests can they be considered scientifically valid. Therefore, like any predictive model, a digital twin must undergo rigorous testing before making far-reaching claims about its capabilities. If significant discrepancies are found, the model must be updated and recalibrated accordingly.

## Clinical benefits of digital twins

From our discussion of the various aspects that digital twins can contribute to P4 medicine, the primary benefits for clinical applications can be summarized as follows:

- Time efficiency
- Cost efficiency
- Ethical
- Time-dependent insights
- Standardization

A particular example illustrating the first four benefits is the selection of a treatment option for a patient. Traditionally, the efficacy of each treatment must be evaluated through randomized controlled trials before being approved for clinical practice. This process is time-consuming, costly, and involves testing on groups of patients. In contrast, the mechanistic models underlying digital twins can be used to optimize treatment plans (duration of administration, number of cycles etc) of FDA approved drugs, when approved treatments do not achieve the desired results. This approach serves as a decision-support system, keeping the doctor in-the-loop for the final decision. It is fast because it can be executed on high-performance clusters, cost-effective as it is performed in silico, and ethical since no living beings are involved. Moreover, digital twins incorporate dynamical aspects of drug effects and provide time-dependent insights into the progression of the patient’s health state.

Lastly, the concept of digital twins provides a systematic approach that is generally applicable to all problems within the P4 medicine framework. This fosters a common language, simplifying the transfer of knowledge across different disciplines. Specifically, a digital twin harmonizes different approaches by integrating diverse data sources, models, and methodologies into a cohesive framework. Through this harmonization, it lays the groundwork for a standardization, enabling consistent and replicable practices across various applications.

### Limitations

Despite their conceptual appeal, digital twins in medicine face fundamental constraints rooted not in technology, but in biology itself. Unlike engineered systems, which are designed to be measured and controlled, the human body is opaque, fragile, and ethically constrained. One cannot embed sensors into every organ or sample tissues continuously. As a result, the ideal of real-time, high-resolution data that is central to engineering digital twins is largely unattainable in clinical settings. Medical digital twins must therefore operate under uncertainty, extrapolate from sparse data, and rely on reasonable approximations. This also means that a complete twin is less important than a strategically useful one. It should be a model that can support clinical decisions even when much of the underlying system remains unobserved.

Moreover, digital twins in medicine are often built on an unspoken metaphor, the idea that a human can be treated like a machine. But unlike machines, humans are shaped by culture, psychology, environment, and randomness in development and disease progression. No digital twin, however sophisticated, can yet capture the full entanglement of mind, body, and context. There is also a risk of overconfidence: clinicians and patients may place too much trust in a DT’s output, mistaking model-based projections for biological certainties. Therefore, beyond technical challenges, the meaningful integration of digital twins into medicine requires philosophical and conceptual clarity. Rather than viewing them as virtual replicas of the patient, medical digital twins should be understood as epistemic companions that serve as tools for exploration, not oracles of truth.

## Conclusion

Just as randomized controlled trials (RCTs) revolutionized evidence-based medicine by establishing a rigorous standard for evaluating treatments, digital twins have the potential to transform the future of personalized medicine and clinical practice. While RCTs serve as the gold standard in clinical research, ensuring the safety and efficacy of interventions across populations, they are inherently limited in addressing individual variability. Digital twins can bridge this gap, providing a dynamic, data-driven model for tailoring interventions at the individual level, much as RCTs have done for population-based care. This is especially relevant in the context of *N*=1 medicine, where digital twins can assist physicians in developing more effective, personalized treatment strategies^25^.

While P4 medicine provides a conceptual foundation with a medical focus, digital twins offer the methodological tools necessary to implement this vision. These two paradigms complement one another, converging at the intersection of patient care. The core capabilities of digital twins—explainability, intervenability, learnability, and diversability—are achieved through the integration of data-driven and model-based approaches. This synthesis deepens our understanding of the dynamic transitions between health and disease. In this light, digital twins can be viewed as learned dynamical systems that can offer valuable insights into time-dependent biological and clinical processes.

## Data Availability

No datasets were generated or analyzed during the current study.
